# Gendered career considerations consolidate from the start of medical education

**DOI:** 10.5116/ijme.5403.2b71

**Published:** 2014-09-13

**Authors:** Margret Alers, Petra Verdonk, Hans Bor, Katarina Hamberg, Antoine Lagro-Janssen

**Affiliations:** 1 Radboud University Medical Center, Department of Primary and Community Care, Gender and Women’s Health Unit, Nijmegen, the Netherlands; 2 VU University Medical Centre, Department of Medical Humanities, EMGO Institute for Health and Care Research, School of Medical Sciences, Amsterdam, the Netherlands; 3 Umea University, Department of Public Health and Clinical Medicine, Family Medicine, Umea, Sweden

**Keywords:** Gender, career, medical education, specialty preference, full-time, part-time, work-life balance

## Abstract

**Objectives:**

To explore changes in specialty preferences and work-related topics during the theoretical phase of Dutch medical education and the role of gender.

**Methods:**

A cohort of medical students at Radboudumc, the Netherlands, was surveyed at start (N=612, 69.1% female) and after three years (N=519, 69.2% female), on specialty preferences, full-time or part-time work, motivational factors, and work-life issues. Chi square tests were performed to analyze gender-differences, and logistic regression to explore the influence of gender on considerations.

**Results:**

A total of 214 female and 78 male students completed both surveys. After three years, the male students remained highly interested in surgery, but the female students increasingly preferred gynecology. These initial preferences were predictive. Four out of five male students versus three out of five female students continued to show a full-time preference. Women increasingly preferred part-time work. After three years, the combination of work, care, and patient contact motivated female students more, whereas salary remained more important to male students. Female students indicated that their future careers would influence their family life; male students assumed having a family would only affect their partners’ careers.

**Conclusions:**

Against an international background of the feminization of medicine, our study shows that career considerations are reinforced early in medical studies. Women prefer to work fewer hours and anticipate care tasks more often. Students’ preferences reflect Dutch cultural norms about working men and women. Therefore, guidance in choice-making much earlier in medical education can create opportunities.

## Introduction

A gendered pattern in specialty preference at the start as well as throughout medical education has been identified in several studies 1-6. Gender differences in medical students’ preferences for specialties are associated with motivational and life-style factors. In addition, cultural values also appear to play a role 7-11. At the start of medical education, female students, for example, anticipate that their career will influence their future family life and they anticipate the combination of work and care, whereas male students are more often motivated by technical skills or career opportunities 1^, ^12. Gender, therefore, plays a role in the development of medical careers from the very start of medical education. Medical education may contribute to students’ motivation for particular specialties. At what stage medical students seriously begin to consider their choice of specialty is unknown. In the first period of medical education, the theoretical content is taught by professionals from the main specialties. As the students get older, thoughts of future relationships and family life may begin to play a role in students’ considerations. Therefore, not only the theoretical content, but also the physicians’ role-modeling, including encouragement or discouragement based on their gender, may influence students in showing particular specialty preferences during this period.

Studies exploring the impact of medical education on gender differences in specialty preferences are scarce. Only Compton et al. 5 give some indication that women remain more interested in primary care (family medicine, internal medicine, gynecology, and pediatrics) during medical education in the US. At an international level, there is an increasing number of women physicians. In the Netherlands, two-thirds of all physicians will be female in 15 years’ time 13-17. To anticipate the future workforce, it is relevant to know male and female students’ career considerations at different stages of medical education and whether these career considerations change over time: How do specialty preferences develop? Do gender differences in specialty preferences exist and do they converge or diverge over the years? And what values, attitudes, and factors are of importance?

We conducted a two-wave longitudinal study at the beginning of year one and at the end of year three in Dutch undergraduate medical education. We explored (1) how specialty preferences of male and female students change, also with respect to related career considerations, such as working hour preferences, motivational factors, and work-life issues, and (2) how predictive initial career considerations are.

## Methods

### Study design

We explored differences between female and male medical students at the Radboud university medical center in the Netherlands, in their considerations regarding specialty preferences and work-related topics: their working hour preferences, ten motivational factors, and eleven work-life issues 1^, ^12. We compared these considerations with their considerations three years earlier at the start of their medical education.

### Participants and procedures

First-year students filled out a printed questionnaire after their very first lecture (2006/2007). Questions in the survey were phrased appropriately so as to measure the content we intended them to measure and were repeatable and consistent. Reliability and validity were thus established. We compared the students’ considerations by surveying the opinions of the same cohort with an identical digital questionnaire at the end of the third year of undergraduate education three years later. This follow-up was based on the students’ special identity number used in medical school. Dutch legislation did not require ethical permission, we did follow the procedures later described by the Ethical Review Board of the Netherlands Association for Medical Education (NVMO) 18. This Review Board was not in place at the time of data collection. Students were informed in advance of the survey that participation was voluntary and that data would be anonymized and treated confidentially.

### Data collection

We collected demographical information on the students’ sex, age, and marital status. The students could choose one of seven main specialties or tick the options ‘other’ or ‘I don’t know.’ Along with the 'other’ option, there was an open space for students to register a free choice of rather small specialties, such as ‘dermatology,’ ‘ophthalmology,’ 'public health,’ ‘pathology,’ or ‘sports medicine.’

The work-related topics we explored, included working hour preferences, motivational factors, and work-life issues. Based on the literature, we defined ten motivational factors that may contribute to the students’ preference for certain specialties, for example, ‘Possibilities for reconciling work and care’ 19. Students indicated the level of importance on a 5-point Likert scale, ranging from ‘not at all’ (1) to ‘completely’ (5). Finally, eleven work-life balance considerations, for example, ‘Do you think that your job and career goals affect your choices in having a family? were assessed on a 5-point Likert scale (totally disagree=1 to totally agree=5).

### Measures

Students who selected more than one answer were categorized in the ‘I don’t know’ group; this was less than 4% of the ‘I don’t know’ group and had an equal male/female ratio.

The numbers of hours students wished to work in the future were grouped into full-time or part-time preference, no paid work, or ‘I don’t know.’ We defined part-time work as less than 36 hours and a doctor’s full-time working week as more than 36 hours, which is more than 0.75 full-time equivalent remunerated work.

### Data analysis

In our analysis, we used a Pearson chi-square test to determine gender differences in our comparison of considerations at the beginning to those at the conclusion of the theoretical stage after three years. We created a dichotomous variable for the motivational factors and work-life issues, as being either with agreement (fully agree or agree) or with disagreement (neutral answer, disagree, and fully disagree), thus clarifying the overall picture. We created a change variable to describe to what degree students adhere to their initial preference or gained or lost this consideration. We used a chi-square to indicate gender differences. Furthermore, we determined the impact of gender and the impact of the initial career consideration on the outcome of the considerations after three years, using a logistic regression model with gender and initial preferences as independent variables. We defined significance at p <.05. Data analysis was performed on SPSS.20 for Windows.

## Results

### Characteristics

A total of 214 female (73.3%) and 78 male (26.7%) students completed both surveys at the beginning and after three years (response rate 56.3%). At the end of the third year, both male and female students were 21.2 years old on average (SD=1.5). Almost half the students were in a relationship at the end of the third year.

**Table 1.** Female and male medical students’ career considerations measured at starts and after three years of study

Variables	Beginning of first year			End of third year		
	Female (n=214)	Male (n-=78)	p	Female (n=214)	Male (n-=78)	p
	% (n)	% (n)		% (n)	% (n)	
Specialty - choosing it						
Internal medicine	7.0 (15)	5.1 (4)	.564	16.0 (34)	22.1 (17)	.227
Psychiatry	3.3 (7)	3.8 (3)	.811	3.3 (7)	6.5 (5)	.226
Neurology	1.9 (4)	1.3 (1)	.732	6.1 (13)	9.1 (7)	.375
Pediatrics	17.3 (37)	9.0 (7)	.079	8.0 (17)	2.6 (2)	.102
Surgery	9.8 (21)	25.6 (20)	.001^*^	5.6 (12)	20.8 (16)	.000^*^
Gynecology	5.1 (11)	(0)	.041^*^	10.3 (22)	1.3 (1)	.012^*^
Family medicine	8.4 (18)	7.7 (6)	.843	15.0 (32)	6.5 (5)	.055
Other	1.9 (4)	5.1 (4)	.131	16.4 (35)	13.0 (10)	.474
I don’t know	45.3 (97)	42.2 (33)	.646	19.2 (41)	26.6 (14)	.838
Working hours						
Full-time ^[Table-fn fn15859]^	48.1(103)	79.5 (62)	.000^*^	33.8 (70)	88.3 (68)	.000^*^
Part-time	50.0 (107)	19.2 (15)	.000^*^	62.3 (129)	11.7 (9)	.000^*^
I don’t know	1.9 (4)	1.3 (1)	.732	3.9 (8)	0	.080
Motivational factors – agreeing						
Interesting content	97.2 (208)	98.7 (76)	.407	99 (202)	98.7 (75)	.615
Career prospects	51.4 (110)	76.6 (59)	.000^*^	43.6 (89)	56.6 (43)	.054
Combination work and care	48.4 (103)	40.3 (31)	.222	60.3 (123)	32.9 (25)	.000^*^
Attractive working hours	36.9 (79)	26.0 (20)	.082	55.4 (113)	42.1 (32)	.048^*^
Lots of direct patient contact	83.2 (178)	75.3 (58)	.131	90.1 (183)	64.5 (49)	.000^*^
Research opportunities	33.0 (70)	49.4 (38)	.011^*^	30.4 (62)	39.5 (30)	.150
Good salary	36.4 (78)	61.0 (46)	.000^*^	28.9 (59)	46.1 (35)	.007^*^
In line with technical skills	38.3 (82)	61.0 (47)	.001^*^	31.4 (64)	48.7 (37)	.007^*^
In line with former work experience	5.6 (12)	5.2 (4)	.892	11.8 (24)	3.9 (3)	.049^*^
In line with former study experience	23.2 (49)	22.4 (76)	.897	36.5 (74)	34.2 (26)	.728
Work-life issues						
you will have equal opportunities as your partner	74.8 (160)	67.9 (57)	.246	68.3 (136)	60.8 (45)	.242
your partner will be less ambitious concerning a career	11.3 (24)	9.1 (7)	.588	18.1 (36)	17.8 (13)	.957
your career affects your choices of having a family	72.9 (156)	73.1 (57)	.976	81.8 (162)	62.2 (46)	.001^*^
having a family affects your career	76.2 (163)	70.5 (55)	.326	77.8 (154)	68.9 (51)	.131
your partner’s career affects your choices to having a family	56.3 (120)	69.2 (54)	.047^*^	58.6 (116)	67.6 (50)	.176
having a family affects your partners’ career	57 (122)	69.1 (50)	.276	48.5 (96)	64.9 (48)	.016^*^
sharing household chores equally between partners	73.8 (158)	55.1 (43)	.002^*^	71 (142)	47.3 (35)	.000^*^
household chores by someone else	22.1 (47)	26.9 (21)	.386	34.7 (69)	37.8 (28)	.627
equal care of your children by yourself and your partner	79 (169)	64.9 (50)	.014^*^	65.8 (131)	64.9 (48)	.882
care for your children by a daycare center	25.7 (55)	25.6 (20)	.992	48.6 (36)	39.4 (78)	.169
care for your children by a nanny, grandparents	57 (122)	53.8 (42)	.690	69.7 (138)	55.4 (41)	.027^*^

^*^p< 0.05

^1^Significant interaction term with gender in preliminary analyses

### Specialty preferences

After three years, surgery remained the second most popular specialty for male students, although they did lose some interest in this specialty (Tables 1 and 2). The influence of gender was very apparent in the preference for gynecology, a specialty that became increasingly popular among female students. An initial preference for gynecology, surgery, family medicine, or not having a preference at all, was highly predictive of students having the same preference considerations at the end of their third year (Table 3).

### Working hour preferences

At the end of their third year, 90% of the male students preferred to work full-time in the future, whereas two-thirds of the female students preferred part-time work. Four out of five male students and three out of five female students who had a full-time preference at the beginning, maintained this preference. One out of four female students had changed their preference from full-time to part-time at the end of their third year. This was rarely the case for male students. Male gender became more predictive of a full-time work preference.

### Motivational factors

For female students, their wish to combine work and care in the future gained importance after the first educational stage. Next to this, female students became more interested in direct patient contact. This contrasted with male students, whose interest in a patient-contact-centering specialty decreased. Male students’ initial higher appreciation of a good salary and technical skills also declined but remained higher than that of female students. Most initial motivational factors were predictive of the same factors at the end of the third year.

### Work-life issues

After three years, female students expected that having a family would influence their future career. For male students, this was not the case. In addition, male students expected that having a family would affect their partners’ careers whereas female students were less likely to expect this. The expected influence of a family on the partners’ career was one of the few work-life issues in which the initial consideration was not influenced after three years.

**Table 2.** Change in female and male students’ career considerations over the first three years of medical education

Changes	No change		Gained interest		Lost interest		p
	Female	Male	Female	Male	Female	Male	
**Specialties** ^*^	% (n)	% (n)	% (n)	% (n)	% (n)	% (n)	% (n)
**Internal Medicine**	1.4 (3)	1.3 (1)	14.5 (31)	20.5 (16)	5.6 (12)	3.8 (3)	.622
**Psychiatry**	0.5 (1)	0	2.8 (6)	6.4 (5)	2.8 (6)	3.8 (3)	.449
**Neurology**	0.5 (1)	0	5.6 (12)	9.0 (7)	1.4 (3)	1.3 (1)	.702
**Pediatrics**	2.8 (6)	0	5.1 (11)	2.6 (2)	14.5 (31)	9.0 (7)	.157
**Surgery**	1.4 (3)	9.0 (7)	4.2 (9)	11.5 (9)	8.4 (18)	16.7 (13)	.000^[Table-fn fn15873]^
**Gynecology**	1.9 (4)	0	8.4 (18)	1.3 (1)	3.3 (7)	0	.024^[Table-fn fn15873]^
**Family medicine**	3.3 (7)	0	11.7 (25)	6.4 (5)	5.1 (11)	7.7 (6)	.173
**Other**	0.5 (1)	2.6 (2)	15.9 (34)	10.3 (8)	1.4 (3)	2.6 (2)	.241
**I don’t know**	12.6 (27)	10.3 (8)	6.5 (14)	7.7 (6)	32.7 (70)	32.1 (25)	.425
**Working hours**	No change		PT -> FT		FT -> PT		
	63.3 (131)	84.4 (65)	12.2 (24)	11.8 (9)	24.0 (47)	2.6 (2)	.000^[Table-fn fn15873]^
**Motivational factors**	No change		Agreed more		Disagreed more		
**Interesting content**	96.1 (196)	97.3 (73)	2.9 (6)	1.3 (1)	1 (2)	1.3 (1)	.727
**Career prospects**	55.4 (113)	65.3 (49)	18.1 (37)	8.0 (6)	26.5 (54)	26.7 (20)	.101
**Combination work and care**	52.2 (106)	69.3 (52)	30.0 (61)	12.0 (9)	17.7 (36)	18.7 (14)	.007^[Table-fn fn15873]^
**Attractive working hours**	53.9 (110)	54.7 (41)	32.4 (66)	30.7 (23)	13.7 (28)	14.7 (11)	.956
**Lots of direct patient contact**	79.8 (162)	57.3 (43)	13.3 (27)	16.0 (12)	6.9 (14)	26.7 (20)	.000^[Table-fn fn15873]^
**Research opportunities**	68.3 (138)	54.7 (41)	14.9 (30)	17.3 (13)	16.8 (34)	28.0 (21)	.073
**Good salary**	69.1 (141)	60.0 (45)	11.8 (24)	13.3 (10)	19.1 (39)	26.7 (20)	.321
**In line with technical skills**	65.2 (133)	61.3 (46)	14.2 (29)	13.3 (10)	20.6 (42)	25.3 (19)	.697
**In line with former work experience**	86.3 (176)	93.3 (70)	10.3 (21)	2.7 (2)	3.4 (7)	4.0 (3)	.819
**In line with former study experience**	63.0 (126)	60.8 (45)	25.0 (50)	24.3 (18)	12.0 (24)	14.9 (11)	.953
**Work-life issues**							
**you will have equal opportunities as your partner**	66.3 (132)	67.6 (50)	13.1 (26)	13.5 (10)	20.6 (41)	18.9 (14)	.953
**your partner will be less ambitious concerning a career**	78.7 (155)	77.8 (56)	13.7 (27)	16.7 (12)	7.6 (15)	5.6 (4)	.726
**your career affects your choices of having a family**	69.7 (138)	60.8 (45)	20.2 (40)	13.5 (10)	10.1 (20)	25.7 (19)	.004^[Table-fn fn15873]^
**having a family affects your career **	72.7 (144)	55.4 (41)	14.6 (29)	21.6 (16)	12.6 (25)	23.0 (17)	.021^[Table-fn fn15873]^
**your partner’s career affects your choices to having a family**	59.9 (118)	56.8 (42)	20.3 (40)	20.3 (15)	19.8 (39)	23.0 (17)	.838
**having a family affects your partners’ career**	56.1 (111)	52.7 (39)	18.2 (36)	24.3 (18)	25.8 (51)	23.0 (17)	.522
**sharing household chores equally between partners**	60.0 (120)	52.7 (39)	18.5 (37)	20.3 (15)	21.5 (43)	27.0 (20)	.521
**household chores by someone else**	65.7 (130)	64.9 (48)	23.7 (47)	24.3 (18)	10.6 (21)	10.8 (8)	.993
**equal care of your children by yourself and your partner**	65.8 (131)	67.1 (49)	10.6 (21)	16.4 (12)	23.6 (47)	16.4 (12)	.246
**care for your children by a daycare center **	66.2 (131)	56.8 (42)	23.7 (47)	32.4 (24)	10.1 (20)	10.8 (8)	.311
**care for your children by a nanny, grandparents**	61.6 (122)	70.3 (52)	25.3 (50)	16.2 (12)	13.1 (26)	13.5 (10)	.277

^1^The change option ‘never’ that fills up to 100% is not in the table.

^*^p< .05,

## Discussion

Our prospective cohort study shows that gendered specialty preferences at the start of medical education are likely to be maintained. Almost all Dutch male students maintain their initial full-time preference, whereas female students switch massively to a preference for part-time work. At the same time 2 out of 3 students are now female. At the same time, the gender gap widens in regard to in regard to expectations of equality in career opportunities. Female students’ initial expectation that they will have equal career opportunities diminishes, while male students increasingly expect that family life will affect the career of their partner but not their own. Female medical students indicate more often that their career will influence their family life, and they become more motivated to choose a specialty that will allow them to maintain a comfortable balance between work and care. At the end of the theoretical stage of their undergraduate medical training, when students enter the clinical stage of medical training, gender plays a more pronounced role in specialty preferences and career considerations than at the beginning.

A fact that is already well-known is that gendered specialty preferences are reinforced during three years of theoretical medical education. An important new finding is that in this early stage, an increasing number of female students prefer part-time work, whereas male students maintain their initial full-time preference. A survey among student members of the Royal Dutch Medical Association shows an even higher percentage of female students wishing to work part-time 20. A Swiss study, however, indicates that working part-time diminishes doctors’ chances of academic success 21. If female students do indeed prefer to work part-time after graduation, and women’s career progress remains hampered by the idea that careers can only be pursued if working full-time, imbalances and shortages of physicians in certain specialties might occur 17^, ^22.

Part-time work can be seen as the result of people’s awareness of a future scenario in which other areas of life are considered to be important, such as leisure time or family life, but also as a hierarchical issue in which working full-time is considered to be more successful 23^, ^24.

**Table 3.** The influence of gender and initial career considerations on the specialty choice after three years^a^

Variables	End of third year			
	Influence of gender		Influence of initial consideration	
	OR (95% CI)	p	OR (95% CI)	p
Specialty				
**Internal medicine**	.67 (.35-1.28)	.223	1.40 (.44-4.45)	.571
**Psychiatry**	.49 (.15-1.60)	.240	2.66 (.31-23.17)	.375
**Neurology**	.64 (.25-1.68)	.364	3.64 (.38-34.45)	.261
**Pediatrics**	2.96 (.66-13.22)	.156	2.58 (.92-7.25)	.073
**Surgery**	.27 (.12-.62)	.002^*^	3.26 (1.33-8.07)	.010^*^
**Gynecology**	7.44 (.98-56.69)	.053	5.84 (1.56-21.88)	.009^*^
**Family medicine**	2.56 (.95-6.89)	.062	3.27 (1.24-8.62)	.017^*^
**Other**	1.41 (.65-3.05)	.382	3.74 (.85-16.55)	.082
**I don’t know**	1.05 (.53-2.08)	.894	2.58 (1.40-4.73)	.002^*^
**Working hours**				
**Full-time**	.08 (.04-.19)	.000^*^	4.09 (2.29-7.29)	.000^*^
**Motivational factors**				
**Interesting content**	1.34 (.12-15.03)	.811	0	.999
**Career prospects**	.66 (.38-1.15)	.146	1.89 (1.15-3.12)	.012^*^
**Combination work and care**	3.11 (1.77-5.47)	.000^*^	1.70 (1.04-2.78)	.036^*^
**Attractive working hours**	1.70 (.99-2.91)	.055	.145 (.87-2.41)	.151
**Lots of direct patient contact**	4.97 (2.56-9.65)	.000^*^	1.61 (.75-3.48)	.226
**Research opportunities**	.82 (.46-1.45)	.484	2.59 (1.53-4.37)	.000^*^
**Good salary**	.59 (.33-1.06)	.076	3.46 (2.03-5.88)	.000^*^
**In line with technical skills**	.60 (.34-1.06)	.077	2.79 (1.67-4.68)	.000^*^
**In line with former work experience**	3.32 (.96-11.48)	.059	4.45 (1.26-15.73)	.021^*^
**In line with former study experience**	1.15 (.65-2.03)	.628	1.64 (.92-2.92)	.096
**Work-life issues**				
**you will have equal opportunities as your partner**	1.77 (.67-4.73)	.253	2.65 (1.52-4.63)	.001^*^
**your partner will be less ambitious concerning a career**	.84 (40-1.78)	.652	2.47 (1.04-5.89)	.041^*^
**your career affects your choices of having a family**	2.25 (.77-6.57)	.138	2.17 (1.17-4.00)	.013^*^
**having a family affects your career**	.57 (.19-1.72)	.321	2.08 (1.14-3.78)	.017^*^
**your partner’s career affects your choices to having a family**	.43 (.16-1.17)	.100	1.73 (1.05-2.86)	.033^*^
**having a family affects your partners’ career**	.35 (.14-87)	.024^*^	1.43 (.87-2.32)	.156
**sharing household chores equally between partners**	3.12 (1.27-7.72)	.014^*^	1.08 (.62-1.88)	.777
**household chores by someone else**	.92 (.48-1.77)	.800	2.47 (1.38-4.42)	.002^*^
**equal care of your children by yourself and your partner**	1.17 (.44-3.11)	.758	2.64 (1.49-4.68)	.001^*^
**care for your children by a daycare center**	.59 (.31-1.11)	.103	2.82 (1.61-4.93)	.000^*^
**care for your children by a nanny, grandparents**	2.82 (1.24-6.42)	.014^*^	2.97 (1.76-5.01)	.000^*^

^1^OR = Odds ratio; 95% CI = Confidence Interval;

^*^p < .05.

Choosing to work part-time could have a cultural dimension: most women in the Netherlands work part-time so as to combine work and care 12^, ^25. Gynecology currently represents the average part-time factor of 0.94 full-time equivalent for men and 0.89 for women, whereas family medicine is the specialty with the highest part-time factor and surgery the one with the highest full-time factor 16. However, the proportion of male and female medical students in a particular specialty is changing, and students’ preferred working hour preference, therefore, will influence future developments.

A major finding in this study is that female students differed from male students in their orientation towards the work-life balance. At this age, students may be more sensitive to signals they receive about family life and the normative values attached to women’s roles within their society. The hidden curriculum may play a role in female and male students’ career preferences. This emphasis in women’s career considerations may reflect the idea of the ‘woman physician’ as a role and the effect of negatively and positively gendered interactions on the evolution of their professional identity 26.

A survey in the US indicated that female physicians (either attending physicians or residents) were even more likely to be the primary childcare providers in the family than women who are not physicians 27 Having a partner at home who takes care of the children allows male physicians to avoid a career break and to work full-time, whilst women make a full-time start, then reduce the number of hours they work after five years and continue to work part-time after that 28. Men in general, however, have been found to be more prepared to accept an egalitarian division of labor than women expect, 29 which may also be true for male physicians in the future. In our study, however, we found that male students mostly expect their partners’ careers to be affected when they have a family of their own.

Our study indicates that female students found patient contact more motivating than male students. This finding is supported by recent studies showing that male students are more extrinsically and female students are more intrinsically motivated 30. Students’ preferences for person-oriented specialties are slightly more likely to be influenced by medical school and less likely to be influenced by income expectations than students’ preferences for technique-oriented specialties 31. As such, different motives for male and female students may influence career considerations early in their studies.

Previous studies have reported gender differences in early specialty preferences, with male students being more interested in surgery and female students in gynecology 6^, ^32. Furthermore, another study showed a partial cohort in which male students with a preference for non- Primary Care specialties, which includes surgery, remained more interested in these specialties than female students, whereas women remained more interested in primary care specialties such as family medicine 5.

### Limitations

In this study, the context of the country in which the study was performed, a context that embraces a cultural family policy in which mothers with young children typically work part-time, may have led to social desirability of the answers students gave. In addition, other experiences during medical education, not measured by us, may have played a role in preference changes: particular role models may have reinforced students’ initial preferences or rather the opposite, and certain disciplines presented in medical education may have been less interesting to students than they initially expected.

Since we found a strong influence of students’ initial full-time preference, further research on the reinforcement of either a career focus or a care focus in medical career considerations is needed. Women may not choose certain specialties because they believe a specialty does not allow doctors to work part-time, whether or not such notions are accurate. It would be interesting to examine the relation between gender and issues in the future work-life balance as these affect the specialty choice of medical students after their clerkships. Understanding how career decisions are made, could give us information about the quality of these decisions and may help to improve the decision-making process.33 In order for students to be aware of any gendered limitations in their career planning process, we would advise them to discuss social roles and discover their talents at an early stage in their medical education.

### Conclusion

During the theoretical part of medical education, gender differences in specialty preferences change as female medical students increasingly tend to attach greater importance to their future work-life balance. As a consequence, they show a higher preference for part-time work and anticipate that their career will have an impact on their future family life. Male students remain focused on full-time work.

Career considerations early on, are highly predictive of career considerations and specialty preferences after the first phase of theoretical medical education. As the students’ preferences reflect Dutch cultural norms about working men and women, there is an opportunity to focus on guidance in choice-making early in medical education.

As two-thirds of the medical undergraduates in our study are female and their ideas about future work-life balance appear to be influencing their career considerations, we recommend raising awareness on career considerations among undergraduate students early on in medical education. Furthermore, attention should be paid to attracting both male and female students to all specialties, to facilitating physicians’ in combining work, leisure, and other obligations, and to supporting initiatives to improve gender equality in family life.
